# Bleeding Phenotype of Glanzmann Thrombasthenia (GT) and Treatment Outcomes in Over One Hundred Patients: A Two-Center Experience in North Pakistan

**DOI:** 10.7759/cureus.73724

**Published:** 2024-11-15

**Authors:** Muhammad Usman, Maryum Khan, Nighat Shahbaz, Lubna Zaffar, Hira Tariq, Raheel Iftikhar, Tariq Ghafoor, Mehreen Ali Khan, Tahira Zafar

**Affiliations:** 1 Clinical Hematology, Armed Forces Bone Marrow Transplant Center, National University of Medical Sciences, Rawalpindi, PAK; 2 Clincal Hematology, Armed Forces Bone Marrow Transplant Center, National University of Medical Sciences, Rawalpindi, PAK; 3 Pathology, Foundation University Medical College, Rawalpindi, PAK; 4 Epidemiology, Armed Forces Bone Marrow Transplant Center, National University of Medical Sciences, Rawalpindi, PAK; 5 Hematology and Oncology, Armed Forces Bone Marrow Transplant Center, National University of Medical Sciences, Rawalpindi, PAK; 6 Pediatrics, Armed Forces Bone Marrow Transplant Center, National University of Medical Sciences, Rawalpindi, PAK; 7 Hematology, Armed Forces Bone Marrow Transplant Center, National University of Medical Sciences, Rawalpindi, PAK; 8 Hematology, Hemophilia Treatment Center, Rawalpindi, PAK

**Keywords:** bleeding disorders, glanzmann thrombasthenia, glanzmann thrombasthenia (gt), hereditary bleeding disorder, one hundred patient, platelets function disorder, thrombocytopathy

## Abstract

Background: Glanzmann thrombasthenia (GT) is a rare disease with an autosomal recessive inheritance pattern. This disorder is not so uncommonly encountered in routine clinical practice and laboratory settings in Pakistan let alone in the rest of the world. To describe the bleeding phenotype of GT and treatment outcomes in over one hundred patients in north Pakistan.

Materials and methods: This descriptive, cross-sectional, retrospective study was conducted on patients from 2011 to 2023 using a convenience sampling technique. A total of 103 patients of all ages and both genders diagnosed as having inherited GT were included in the study.

Results: The median age of the study population was 1.1 years, with an interquartile range (IQR) of 0.8-2. Out of the total, 55 (53%) patients were males and 48 (47%) patients were females. Ninety-eight percent of patients were diagnosed using light transmission aggregometry, and only two (2%) patients were diagnosed by immunophenotyping. Due to the high incidence of interfamily marriages, 86 (84%) patients were born to consanguineous marriages. Thirty-nine (38%) patients had an episode of major bleeding as defined by the International Society on Thrombosis and Haemostasis (ISTH) criteria. Epistaxis in 73 (71%) patients, skin bruising in 63 (61%), and gum bleeding in 57 (55%) were the most common bleeding symptoms. Thirty-two (31%) required the use of r-VIIa for major bleeding and five (5%) patients underwent fully matched allogeneic HSCT (hematopoietic stem cell transplant). Graft versus host disease-free relapse-free survival (GRFS) was 80%.

Conclusion: GT is still an underrecognized and underdiagnosed disorder, particularly in resource-limited settings where the estimated incidence seems to be much higher than reported.

## Introduction

Glanzmann thrombasthenia (GT) is a rare disease with an autosomal recessive inheritance pattern. This disorder was first discovered by Swiss pediatrician Eduard Glanzmann in 1918 [1]. It is caused by quantitative or qualitative deficiency of alpha IIb beta3 platelet integrin resulting in impaired platelet function and bleeding diathesis [2,3]. This disorder has three types: type 1, characterized by <5% normal glycoprotein (GP) IIb/IIIa expression, and type 2 with 5-20% normal GP expression. Type 3 is characterized by normal expression of GP but impaired function [4]. The exact incidence of the disorder is unknown, but it is higher in certain ethnic groups [5]. A careful history and appropriate investigations are essential for early diagnosis of this disease to avoid delays in diagnosis. Light transmission aggregometry is the gold standard for the diagnosis of GT; however, it is time-consuming and difficult to perform in children [6]. Other screening tests, including thromboelastogram (TEG), may be used as an initial screening tool in certain settings [3]. Whole multiple electrode aggregometry (MEA) or flow cytometry has also been shown to sensitively detect GT [7,8]. Platelet transfusions are usually avoided unless the bleeding is unresponsive to other measures, including tranexamic acid and r-VIIa, partly due to the risk of alloimmunization or concerns for lack of efficacy of transfused platelets due to diseased platelets adhering but not functioning [9,10]. GT is considered a rare disorder with a worldwide incidence of approximately one in 1 million [11]. This disorder is often seen in routine clinical practice and laboratory settings in Pakistan [12], let alone in the rest of the world. We now report our experience in one of the largest concentrations of GT patients at two centers.

## Materials and methods

Study design and sampling technique

This descriptive, cross-sectional, study was conducted on patients registered with the Hemophilia Treatment Center (HTC), Rawalpindi, and presented to the Armed Forces Bone Marrow Transplant Center (AFBMTC), Rawalpindi from 2011 to 2023 using a convenience sampling technique. The work described has been conducted per the code of ethics of the World Medical Association by the Declaration of Helsinki for experiments involving humans. The Armed Forces Bone Marrow Transplant Center IRB Committee issued approval IRB-030/AFBMTC/Approval/2022. 

Inclusion and exclusion criteria

A total of 103 patients of all ages and both genders diagnosed as having inherited GT were included in the study. Patients with acquired thrombasthenic caused by autoimmune disorders or drugs were excluded.

The patients were diagnosed by either light transmission aggregometry (LTA), showing impaired responses to all agonists except ristocetin, or immunophenotyping, showing absent or reduced expression of CD41 and CD61 on platelets.

Statistical analysis

In descriptive analysis, percentage and frequency were calculated for categorical variables and median, interquartile range (IQR), and range for all the continuous variables. The Kaplan-Meier test was applied to check overall survival and disease-free survival.

## Results

The study group comprised 103 patients with a diagnosis of GT. The median age of the study population is 1.1 years with IQR of (0.8-2) range (0.02 to 17 years). Out of the total, 55 (53.3%) patients were male and 48 (46.6%) patients were female. Ninety-eight percent of patients were diagnosed using light transmission aggregometry and only two (1.9%) patients by immunophenotyping. Due to the high incidence of interfamily marriages, 86 (84%) patients were born to consanguineous marriages, and 59 (57.2%) had a positive family history of bleeding disorders in 1st-degree relatives. Among the patients, 39 (38%) had an episode of major bleeding as defined by the International Society on Thrombosis and Haemostasis (ISTH) criteria [13]. Epistaxis in 73 (71%) patients, skin bruising in 63 (61%), and gum bleeding in 57 (55%) were the most common bleeding symptoms (Table 1). Among male patients, four (7%) had post-circumcision bleeding.

**Table 1:** Bleeding manifestations

Bleeding manifestations	n	%
Umbilical stump bleeding	2	2
Bruising	63	61
Ear bleeding	4	4
Epistaxis	73	71
Gum bleeding	57	55
Dental bleeding	32	31
CNS bleeding	0	0
Joint bleeding	1	1
Gastrointestinal bleeding	7	7
Hematoma	2	2
Hematuria	1	1
Post-trauma bleeding	9	9
Post-surgery bleeding	4	4

None of the patients had CNS (central nervous system) bleeding. Gastrointestinal (GI) bleeding was seen in seven (7%) patients. One patient had severe GI bleeding, angiodysplasia of the colon, and severe platelet refractoriness. He had episodes of massive hemorrhage and required a massive transfusion protocol, recombinant FVII concentrates, and ultimately angioembolization. Although this patient required mechanical ventilation during the episode of bleeding, he was ultimately weaned off and discharged home. Presently he is on recombinant VII (r-VII) weekly prophylaxis and has occasional episodes of bleeding from the nose or gums requiring breakthrough factor VII treatment. He underwent fully matched HSCT with his sister and is three months post-transplant at the time of writing this manuscript and has full donor chimerism and has no major post-transplant complication.

Iron deficiency was universally present in all patients with GT and was diagnosed by either characteristic hypochromic microcytic blood picture or serum ferritin less than 30 ng/ml. All patients were given concomitant oral iron replacement at the time of diagnosis. Around 50% of patients received red cell concentrates (RCC) which was either due to severe iron deficiency anemia or acute hemorrhage. Data regarding what percentage of patients exactly received RCC for severe iron deficiency anemia alone was not retrievable from records.

Of 20 adolescent females, menorrhagia occurred in 12 (60%) and all of them were advised to use tranexamic acid till the resolution of symptoms. Hormone supplementation for control of menorrhagia was required in all patients with menorrhagia. All patients had a reduction in heavy menstrual bleeding (HMB) following these measures and none of them required further interventions like blood product transfusion, hospitalization, use of r-VII, or any surgical procedure for control of HMB. Data regarding the reduction in HMB was retrieved from physician notes. Formal assessment methods assessing reduction in HMB were not utilized. Reduction in the number of sanitary pads used, reduced passage of clots, and patient self-reported reduction in bleeding symptoms were primarily used as response assessment criteria. Only three (15%) female patients eventually were married and there was one (33%) pregnancy complicated by post-partum hemorrhage, requiring massive transfusion protocol and r-VIIa along with platelet transfusion.

Fifty-one (50%) patients received RCC transfusions either due to the consequence of major hemorrhage or severe iron deficiency anemia. Platelet transfusions were given in 37 (36%) of patients with WHO Grade 2 or above bleeding not responding to tranexamic acid and where r-VIIa concentrates were not available. Among patients, 32 (31%) required the use of r-VIIa either for major bleeding, unresponsive to platelet transfusion, or in front-line settings with no cost constraints. Due to the non-availability of human leukocyte antigen (HLA)-matched platelets and resource constraints, random donor platelet transfusions were used in 37 (36%) patients with GT in major bleeding. Anti-platelet alloantibody testing is not available countrywide, so patients with persistence of bleeding despite platelet transfusion were considered platelet refractory and offered r-VIIa in case of clinically significant bleeding. Data regarding which of these patients had platelet allo reactivity are missing. Of the total patient population, five (5%) patients underwent fully matched allogeneic HSCT. The conditioning regimen used for these patients is Bu12.8, Cy120, and ATG 20. Anti-thymocyte globulin (ATG) was added due to the potential risk of the presence of alloantibodies in this patient population due to pre-transplant random donor platelet transfusion (Table 2).

**Table 2:** Description of treatment

	n	%
Tranexamic acid	94	91
Red cell concentrate	51	50
Fresh frozen plasma	44	43
Platelets transfusion	37	36
Recombinant-VIIa	32	31
Bone marrow transplant	5	5

**Figure 1:**
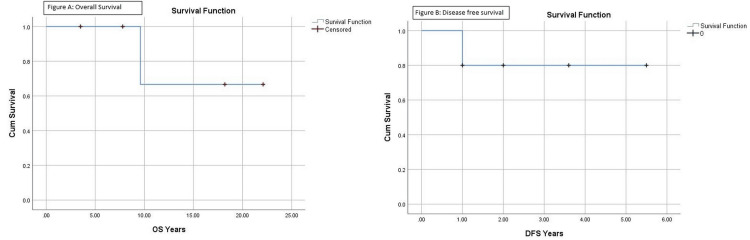
(A) Overall survival and (B) disease-free survival OS: overall survival; DFS: disease-free survival

Out of five allogeneic HSCT patients, the overall survival (OS) rate was 80% with a mean of 18 years of survival; disease-free survival (DFS) was 80% with a mean of 4.6 years of survival (Figures 1A, 1B); graft versus host disease-free relapse-free survival (GRFS) was 80%; and one patient had severe GVHD (graft-versus-host disease) of eyes and lungs. There was no mortality in the non-transplant arm due to bleeding-related complications.

## Discussion

This study presents real-world data from a large cohort of patients presenting with GT in northern Pakistan and highlights bleeding patterns and severity of this patient population along with challenges in the management of these patients. To the best of our knowledge, this is the largest biocenter study of this otherwise rare thrombasthenia reported from northern Pakistan. Another study from a reference diagnostic center in northern Pakistan had an even larger number, but the study spanned over 12 years [11,12]. Our cohort of 103 patients showed a slight predominance of male patients compared to female patients, which is in keeping with previously published data [13-15]. A very large study from Pakistan showed a higher predominance of female patients [12]. The median diagnostic age of our patient cohort is 1.1 years (1-4 years) (0.8- 2), which highlights the earlier presentation of this disease in children under five years of age [16]. However, some studies have shown delayed age of diagnosis, which could be due to failure of recognition of this disorder in early years or presentation in later life, e.g., isolated menorrhagia in females as first presentation [17]. GT is a recessively inherited benign bleeding disorder with higher prevalence in areas where consanguineous marriages are common [18-20]. Although the national registry is run by the Hemophilia Foundation of Pakistan, the presence of a positive family history for bleeding disorders and consanguinity in more than 50% of the patient population suggests increased incidence in our population due to its mode of inheritance. Light transmission aggregometry was almost exclusively used for the diagnosis of GT in our patients, and only 2% of patients were diagnosed using flow cytometry [21]. We did not perform immunophenotyping alongside LTA and relied on a single diagnostic modality, although discrepant results between immunophenotyping and LTA may be seen in acquired GT, leukocyte adhesion defects, and type 3 GT [22].

The bleeding pattern in our patient cohort is presented in Table 1. Although the genetic and ethnic correlation of GT with bleeding manifestation has been suggested in case reports, no clear pattern has been identified so far [17]. Epistaxis is often the most common bleeding manifestation in GT, which occurred in around 70% of patients and was seen in a similar proportion of our patient population [22]. HMB is another main area of concern and was seen in 60% of female patients, which was probably an underestimation as the pictorial blood chart assessment was not implemented [23]. HMB severely affected their quality of life in terms of multiple hospital admissions and blood transfusions. Post-surgery and post-trauma bleeding was seen in less than 10% of patients [24]. The reason for this discrepancy could be due to our multidisciplinary care program entailing patient education, lack of 24-hour telephonic access in case of emergency, and personal contacts of hematologists with expertise in bleeding disorders with anesthetists, surgeons, obstetricians, dentists, blood bank physicians, and other specialties. Intuitively, this leads to appropriate and timely intervention and, thus, a lower risk of post-surgical and post-trauma bleeding despite the limited availability of r-VIIa and the non-availability of platelet antibody status [24]. Detailed hemostatic cover plans are provided for surgeries to ensure proper care of these patients. Around 30% of patients experienced major hemorrhage in GT, which is lower than previously described in some older studies [17]. This could be due to better supportive care over time and earlier recognition of hemorrhage. GT is shown to be associated with a more severe bleeding phenotype than other congenital platelet function defects in a large study [25]. Bruising, epistaxis HMB, post-partum, and post-operative bleeding were the most common bleeding manifestations [25,26].

In our study, post-circumcision and umbilical stump bleeding were uncommon and these are historically rare bleeding sites in GT [20]. The disorder is usually characterized by mild mucocutaneous bleeding and deep organ and musculoskeletal and joint bleeding are rare as highlighted by our cohort as well [27]. Similarly, no patient in our cohort had CNS bleeding and it again is a very rare bleeding site in GT [28]. Prophylaxis with r-VIIa has been given in one patient only, who had multiple episodes of severe life-threatening GI bleed as described above and this strategy has been employed rarely [29]. Gastrointestinal bleeding including severe GI bleeding was observed in our patient cohort and was managed with r-VII, platelet transfusions. Random or single donor platelet transfusions were used in 36% of our patients as HLA-matched platelets are rarely available and testing for anti-HLA and anti-HPA (human platelet antigen) antibodies is not done in Pakistan due to the huge cost of testing [30]. In major bleeds, irrespective of the bleeding site, r-VIIa was given in 31% of patients [31-33]. All patients except one responded to it.

Iron deficiency anemia secondary to chronic blood loss was universally present in all our patients and routine oral iron supplementation was given to all of them. Red cell concentrate transfusions were given in 50% of our patients either due to severe acute hemorrhage or severe anemia secondary to ongoing blood loss when parenteral iron replacement alone was considered insufficient. Since platelet transfusion for major hemorrhage is not as effective in GT as some other thrombasthenias [34,35] ongoing hemorrhage often requires RCC transfusions to prevent acute severe anemia [35]. Around 42% of patients also received fresh frozen plasma (FFP) for an episode of bleeding which is not routinely recommended in patients with GT but has been used in desperate attempts in severe hemorrhage. Most of these transfusions were done in hospitals and clinics located in peripheral and rural areas of Pakistan where facilities for platelet transfusions were not available. Although all patients with GT were given emergency contact numbers of the hemophilia treatment center (HTC) and written plans in case of emergency bleeds, FFP transfusion was still administered in these rural hospitals. This highlights a lack of awareness among general practitioners and other personnel about the management of inherited bleeding disorders and the hazards of unnecessary blood product transfusion. Data regarding its efficacy is lacking and its use should be discouraged in GT [12]. This cohort shows how females diagnosed with this disorder rarely get married in underdeveloped countries due to the stigma associated with bleeding disorders in young girls and women [36]. The taboo around excessive menstruation causes these girls and women to become socially isolated thus resulting in poor social relations. This needs to be addressed at a national level to increase awareness about menstrual health in female patients with all bleeding disorders to ensure these patients live as near normal lives as possible and enjoy sexual intimacy and matrimony [37]. Likewise, there were reports of school-going children missing school due to bleeding episodes but the exact number of absentees could not be retrieved from records.

The limitations of our study include its retrospective nature and missing data, particularly on the quantitative bleeding assessment of HMB. Also, there was a lack of testing for allo-immunization in GT patients. Patients with severe phenotypes who were referred to a transplant center early underwent allo-HSCT; however, the eligibility of allo-HSCT was assessed based on physician assessment of severe bleeding. The eligibility criteria for allo-HSCT need to be standardized and bleeding severity assessment needs to be done as per validated and standardized criteria [38].

## Conclusions

This study highlights the real-world scenario of bleeding patterns and treatment outcomes of GT from the two largest hematology centers in Pakistan. GT is still an under-recognized and under-diagnosed disorder, particularly in resource-limited settings where the estimated incidence seems to be much higher than reported. Devising bleeding assessment tools validated for platelet function disorders would probably lead to earlier detection of these patients. Also, national-level attention should be paid to these disorders to increase awareness among the masses and medical professionals to improve the care of persons with bleeding disorders.
